# Dentin microhardness changes and smears layer removal following conventional versus alternative irrigation regimens in root canals

**DOI:** 10.6026/973206300223740

**Published:** 2026-06-30

**Authors:** Shefali Talekar, Adiya Apon, Sarita Vikram Singh, Smitha Reddy, Prachi Kapade, Ritika Pundir, Miral Mehta

**Affiliations:** 1 Department of Conservative Dentistry and Endodontics, Bharati Vidyapeeth Dental College and Hospital, Navi Mumbai, Maharashtra, India; 2 Department of Dentistry, Nagaland Institute of Medical Sciences and Research, Kohima, Nagaland, India; 3 Department of Conservative Dentistry and Endodontics, Bharati Vidyapeeth (Deemed to be University) Dental College and Hospital, Pune, Maharashtra, India; 4 Department of Conservative Dentistry and Endodontics, Sri Sai College of Dental Surgery Vikarabad, Telangana, India; 5 Department of Oral Pathology and Microbiology, MGV's KBH Dental College and Hospital, Nashik, Maharashtra, India; 6 Department of Conservative Dentistry and Endodontics, NSVK Shri Venkateshwara Dental College and Hospital, Bangalore, Karnataka, India; 7 Department of Pediatric and Preventive Dentistry, Karnavati School of Dentistry, Karnavati University, Gandhinagar, Gujarat, India

**Keywords:** Root canal irrigation, dentin microhardness, smear layer, sodium hypochlorite, ethylenediaminetetraacetic acid (EDTA), QMix, MTAD, scanning electron microscopy (SEM)

## Abstract

Effective irrigation during root canal treatment is essential for disinfection and smear layer removal, through its effects on dentin microstructure remain insufficiently explored. Therefore, it is of interest to compare the five irrigation protocols-NaOCl/EDTA, NaOCl/ citric acid, CHX/EDTA, MTAD and QMix-on dentin microhardness and smear layer removal. All experimental groups showed significant microhardness reduction compared with control, with NaOCl/EDTA causing the greatest decrease. QMix and MTAD demonstrated superior smear layer removal with moderate effects on dentin hardness, while QMix showed consistent performance across root levels. Thus, data shows the QMix and MTAD provide a balanced alternative to conventional NaOCl/EDTA, maintaining effective smear-layer removal while reducing the negative impact on dentin microhardness.

## Background:

The efficient chemomechanical debridement of the root canal system, including removal of pulp tissue remnants, microorganisms and the amorphous smear layer inevitably produced during mechanical instrumentation, is a key element of successful root canal treatment [1]. The smear layer, which was initially outlined in the endodontics field during the early 1980s, is a coating of roughly 1-2 μm thick consisting of organic and inorganic components such as dentin chips, pulp tissue fragments, odontoblastic processes, bacteria and bacterial byproducts, which is burnished onto the instrumented canal walls [2]. This layer blocks the dentinal tubule orifices, which may contain microorganisms in their matrix and which may block the adaptation and penetration of intracanal medicaments, sealers and obturation materials to the dentinal tubular system [3]. Elimination of the smear layer followed by obturation has been popular in modern endodontic practice and this has been shown to result in improved sealer penetration, better dentinal tubule penetration, lower bacterial harboring and long-term sealing capacity in cases where root canal surfaces are not contaminated with smear layer [4]. The traditional irrigation procedure for removing the smear layer uses sodium hypochlorite (NaOCl) to dissolve the organic portion of the smear layer, followed by ethylenediaminetetraacetic acid (EDTA) to dissolve the inorganic constituent. This combination has served as the gold standard in endodontic irrigation for decades [5]. Endodontic Sodium hypochlorite (0.5-6 percent) is the most widely used root canal irrigant due to its strong antimicrobial effect, its ability to dissolve organic tissue and its cost-effectiveness [6]. Its disadvantages, however, are well documented, such as cytotoxicity to periapical tissue when extruded, unfavourable taste and odour, the possibility of discolouration of tooth structure and the inability to remove the inorganic part of the smear layer alone [7]. EDTA, which is normally applied as a 17 per cent aqueous solution, is an agent that dissolves calcium and demineralizes the inorganic dentin matrix, thereby allowing smear-layer dissolution [8]. The combination of the two solutions in sequence is the foundation of the traditional endodontic irrigation procedure used worldwide. There is increasing evidence of the harmful effects of traditional irrigation procedures on the mechanical and structural characteristics of radicular dentin. The chelating and demineralizing effects of sodium hypochlorite, as well as its proteolytic effect, may alter the calcium/phosphorus ratio, the integrity of the organic matrix and, eventually, microhardness and fracture resistance of root dentin [9]. These changes have special clinical importance because endodontically treated teeth are prone to fracture by definition and further loss of dentin structural integrity can negatively impact the tooth's long-term survival [10]. To counter these fears, alternative irrigant solutions and combinations have been established and explored. Citric acid, at concentrations of 10 to 50 percent, has been suggested as a replacement for EDTA, which has a similar chelating action and may have varying effects on the dentin substrate [11]. As a substitute or in combination irrigant, chlorhexidine gluconate (CHX), a broad-spectrum antimicrobial agent, has been proposed because of its substantial antimicrobial activity, reduced cytotoxicity profile and the absence of tissue-dissolving properties compared with sodium hypochlorite [12]. The introduction of MTAD, a combination of doxycycline, citric acid and a detergent (Tween 80), is an all-encompassing final irrigation solution that can combat both organic and inorganic smear-layer elements and is also antimicrobial [13]. A relatively new commercially available QMix was developed as a single-solution final irrigant, with an EDTA, chlorhexidine and surfactant formulation, or, in other words, a combination of chelating, antimicrobial and wetting properties in a single formulation [14]. Although components of these irrigant solutions have been analyzed individually, a comparative analysis of their action in relation to the dentin microhardness variation and the efficacy of the smear layer as the outcome of various routine as well as alternative irrigation protocols in a single, standardized experimental design have been scarcely conducted in the literature [15]. Moreover, a significant proportion of available evidence has considered either microhardness or the removal of the smear layer in isolation, rather than as a prospective measure of both parameters, which is necessary to define clinical trade-offs between cleaning efficacy and structural preservation [16]. Therefore, it is of interest to compare the effects of five irrigation regimens comprising NaOCl/EDTA, NaOCl /citric acid, CHX/EDTA, MTAD and QMix on radicular dentin microhardness and smear layer removal efficiency at the varying levels of root canals and dentin depths.

## Materials and Methods:

## Design of the study and selection of the sample:

This comparative experimental study was conducted in vitro at the Department of Conservative Dentistry and Endodontics in partnership with the Central Research Laboratory. The 100 freshly extracted single-rooted human premolars were extracted for orthodontic or periodontal reasons, with the patients' informed consent and institutional ethical approval. The inclusion criteria in terms of tooth selection included: single-rooted, single straight root (using the method of Schneider) and no root caries or external root resorption, having no previous endodontics and showing no visible root cracks or fractures at 10x magnification and with a minimum root length of 14 mm after decoronation. The exclusion criteria were: teeth with calcified canals or internal resorption, teeth with more than one canal, teeth with a canonical root curvature of over 10 degrees, teeth with developmental defects and teeth kept for more than 3 months after extraction. After extraction, the entire teeth were debrided of soft tissue debris and calculus, as well as pieces of periodontal ligament, using hand scalers and kept in a 0.1% thymol solution at 4°C until use (maximum storage: 8 weeks).

## Specimen preparation:

A uniform, continuous water-cooling was used to decoronate all the teeth at the cementoenamel junction with a slow-speed diamond disc to obtain standardized root segments 14 mm long. A size 10 K-file was inserted into the canal until the tip appeared at the apical foramen and the length was calculated by subtracting 1 mm. Canal patency was established by canal inspection in every specimen. A master apical file size of F3 (apical preparation diameter of 0.30 mm) was achieved by instrumenting root canals with a crown-down technique with ProTaper Gold rotary nickel-titanium files (Dentsply Sirona, Ballaigues, Switzerland). Instrumentation was performed using 2 mL of normal saline introduced between each file to wash away all gross debris, irrespective of the group used. The last irrigation procedure in each experimental group was performed once instrumentation was achieved.

## Group allocation and irrigation protocols:

The specimens were randomly assigned to six groups with the help of a computer-generated randomization table:

[1] Group 1 - NaOCl/EDTA (Conventional Protocol, n=16): 5 mL of 5.25% NaOCl was washed over the sample for 3 minutes, 5 mL of 17% EDTA was transferred over the sample for 1 minute, followed by 5 mL of 5.25% NaOCl washed over the sample for 1 minute, followed by 3 mL of distilled water flushed across sample.

[2] Group 2 - NaOCl/Citric Acid (n=16): Final addition of 5mL of 5.25% NaOCl after 3 minutes, addition of 5mL of 10% citric acid after 1 minute and then 5mL of 5.25% NaOCl, followed by a final flush of 3mL of distilled water.

[3] Group 3 - CHX/EDTA (n=16): 3 minutes of final irrigation with 5 mL of 2% chlorhexidine gluconate, 1 minute of final irrigation with 5 mL of 17% EDTA, 1 minute of final irrigation with 5 mL of 2% chlorhexidine and a terminal flush with 3 mL of distilled water. The flushing between CHX and EDTA applications, with a large volume of intermediate saline, was conducted to ensure no precipitate formed.

[4] Group 4 - MTAD (n = 16): Irrigation with 5 mL of 1.3% NaOCl (3 minutes) during the last instrumentation procedure, final rinse, 5 mL of BioPure MTAD (Dentsply Tulsa Dental, Tulsa, OK, USA) (5 minutes) as instructed by the manufacturer and terminal rinse with 3 mL of distilled water.

[5] Group 5 - QMix (n = 16): Add 3 mL of 5.25% NaOCl and 90 s of QMix 2in1 (Dentsply Tulsa Dental), then finalize with 3 mL of distilled water.

[6] Group 6 - Saline Control (n=20): Final irrigation of 15 mL sterile normal saline administered during 5 minutes. The entire irrigant solutions were applied using 30-gauge side-vented needles (NaviTip, Ultradent Products, South Jordan, UT, USA) inserted to a depth of about 2 mm of the working length at a constant flow rate of about 1 mL/15 seconds. All solutions were maintained at room temperature (23 ± 1oC). Each specimen apical foramen was covered with sticky wax to prevent extrusion of an irrigant and to model a closed-end canal system.

## Smear layer assessment- Scanning electronic microscopy:

One out of every eight samples of each of the experimental groups and one out of every ten samples of the control group were randomly chosen to undergo smear layer assessment. All the roots were longitudinally grooved on the buccal and lingual surface with a diamond disc without piercing the canal lumen and two halves were made with a chisel and mallet. The most clearly visible canal wall was picked and analyzed. The specimens were fixed in 2.5 percent glutaraldehyde overnight, then dried in ascending concentrations of ethanol (25 percent, 50 percent, 75 percent, 95 percent, 100 percent) for 20 minutes each, dried with hexamethyldisilazane, glued to aluminum stubs and sputter-coated with gold-palladium (20 nm thick). A field emission scanning electron microscope (JSM-7600F, JEOL and Tokyo, Japan) was used for scanning electron microscopy (SEM) at an accelerating voltage of 15 kV. Standardized locations were taken in representative photomicrographs at 1000 magnification at three different positions: coronal third (9 -11 mm at apex), middle third (5 -7 mm at apex) and apical third (1 -3 mm at apex). The smear layer injury was assessed as an independent variable between two calibrated examiners who were not aware of the group assignment and applied a five-point scoring system, which was previously tested and found valid:

[1] Score 1: No smear layer; tubules open and clean.

[2] Score 2: Slim layer of smear; most of the tubules are open.

[3] Score 3: Moderate smear layer; about 50% of the tubules open.

[4] Score 4: Thick smear layer; a minimal number of tubules are seen.

[5] Score 5: Full coverage of the smear layer; no tubules can be seen.

The inter-examiner calibration was assessed using a pilot group of 20 photomicrographs, independently, with a Cohen's kappa of 0.86.

## Dentin microhardness testing:

The other eight specimens from each experimental group and ten specimens from the control group were selected for examination of hardness. The roots were placed in self-cure acrylic resin and cut transversely at three levels with respect to the apex (10 mm, 6 mm and 2 mm, respectively) with a precision low-speed saw (IsoMet 1000, Buehler, Lake Bluff, IL, USA) with constant water flow to yield sections with a thickness of about 2 mm. Each section was polished sequentially with silicon carbide abrasive papers of 600, 800, 1200 and 2000 grit under water lubrication, followed by polishing the end of the coronal surface with a 0.3-0.3-0.3-grit alumina suspension on a felt disc to a mirror-like finish. Vickers microhardness testing was conducted using a digital microhardness tester (HMV-2T, Shimadzu Corporation, Kyoto, Japan) with a diamond Vickers indenter, applying a load of 50 gf (0.49 N) for 15 seconds. Each section had three indentations at a distance of 500 μm above the canal lumen and three more indentations at a distance of 1000 μm above the canal lumen, which were located at the same distance around the canal circumference. The mean of three measurements at each depth was used as the representative Vickers hardness number (VHN) for that point.

## Statistical analysis:

SPSS (version 26.0, IBM Corporation) was used to analyze all data. The Shapiro-Wilk test was used to confirm the normal distribution of the microhardness data. Microhardness results were analyzed using three-way ANOVA (factors: irrigation group, root level, measurement depth) and Tukey's HSD post hoc test for multiple comparisons. The Smear layer scores, which are ordinal data, were compared using the Kruskal-Wallis test to assess overall group differences. The Kruskal-Wallis test was not applicable to analyze pairwise group comparisons; these were analyzed using the Mann-Whitney U test with a Bonferroni correction. The Friedman test was used to make intra-group comparisons at different levels of roots. The p-value was determined to be significant at p = 0.05.

## Results:

Microhardness values measured at 500 μm from the canal lumen demonstrated statistically significant differences among the irrigation group's at all three root levels (p < 0.001). The saline control group exhibited the highest mean microhardness values across all root levels, confirming that instrumental preparation alone does not significantly alter dentin hardness at this depth when no chemically active irrigant is used. Among the experimental groups, Group 1 (NaOCl/EDTA) produced the most pronounced microhardness reduction at all levels, followed closely by Group 2 (NaOCl/citric acid). Groups 4 (MTAD) and 5 (QMix) exhibited intermediate microhardness values that were significantly higher than those of Groups 1 and 2 but significantly lower than the control. Group 3 (CHX/EDTA) exhibited microhardness values intermediate between the NaOCl-containing groups and the alternative-irrigant groups (Table 1). Microhardness values measured at 1000 μm from the canal lumen also revealed significant inter-group differences, although the magnitude of reduction was less pronounced than at 500 μm. The same rank order among groups was maintained, with Groups 1 and 2 showing the greatest reduction and Groups 4 and 5 demonstrating the most favorable preservation of microhardness. Notably, at the 1000 μm depth, the differences between the alternative irrigant groups (MTAD and QMix) and the saline control group did not reach statistical significance in the coronal third, suggesting that the demineralizing effect of these solutions has a more limited depth of penetration compared to the conventional NaOCl/EDTA regimen (Table 2). Smear layer evaluation demonstrated significant differences among groups at all root levels (p < 0.001). The saline control group exhibited the highest (worst) smear-layer scores across all thirds, confirming that saline alone is ineffective for smear-layer removal. Among the experimental groups, Group 5 (QMix) achieved the lowest median smear-layer scores across all root levels, indicating the most effective and consistent smear-layer removal. Groups 1 (NaOCl/EDTA) and 4 (MTAD) performed comparably, with no significant differences between them at any root level. Group 2 (NaOCl/citric acid) demonstrated effective smear layer removal in the coronal and middle thirds but was significantly less effective in the apical third compared to Groups 1 and 5. Group 3 (CHX/EDTA) showed acceptable performance in the coronal third but inferior scores in the middle and apical thirds. All groups demonstrated progressively less effective smear-layer removal from the coronal toward the apical third (p < 0.05 for intra-group comparisons) (Table 3).

## Discussion:

The results of this paper reveal that the irrigation regimen used in root canal treatment has a highly variable impact on the mechanical properties of radicular dentin and on the effectiveness of smear layer removal. Therefore, the fundamental trade-off between aggressive chemical debridement and the maintenance of tooth structural integrity is inherent to this procedure. QMix and MTAD turned out to be the most balanced protocols among the tested ones, as they provide efficient smear-layer removal with a significantly smaller impact on microhardness loss than the traditional NaOCl/MTAD protocol. The marked decrease in microhardness observed with the use of the NaOCl/EDTA combination can be attributed to the synergistic and sequential action of these chemically distinct solutions on the organic and inorganic components of dentin. EDTA removes calcium ions from hydroxyapatite crystals, disrupting the mineral framework that comprises about 70 percent of dentin by weight. In contrast, the proteolytic effect of sodium hypochlorite disrupts the collagen matrix within which mineral deposition takes place [17]. Past studies have consistently shown that the interaction of these two agents causes more dentin softening than when each solution is appliedalone and exposing the dentin to the two agents sequentially further enhances the demineralization effect, which is attributed to a mechanism of alternating mineral and organic matrix loss [18]. The current results, which indicate that the microhardness decreases by about 33-38 at a depth of 500 μm in the NaOCl/EDTA sample, are nearly in agreement with existing data from comparable methodology studies [19]. The NaOCl/citric acid mixture showed microhardness changes and behavioral patterns similar to those of the NaOCl/EDTA protocol, except that the coronal and middle thirds exhibited slightly weaker effects. Instead of acting as an ionic chelating agent, citric acid acts as an organic acid that dissolves calcium in hydroxyapatite by acid dissolution and not ionic chelation. This reaction could result in a somewhat variable pattern of mineral loss in the dentin substrate [20]. Nevertheless, the similar general decrease in microhardness across these two protocols indicates that the ultimate biologic task of mineral removal is biologically equivalent, whether it is due to the specific chemical reaction occurring, with clinical implications for the substitutability of these chelating solutions [21]. The much higher microhardness values observed under the CHX/EDTA protocol than under the NaOCl-containing protocols can be directly attributed to the non-proteolytic chlorhexidine. In contrast to sodium hypochlorite, chlorhexidine cannot dissolve organic tissue and thus does not dissolve the collagen matrix of dentin [22]. The observed decrease in microhardness in this group is thus mainly due to the chelating effect of EDTA, rather than to the compounding effect of nitration of the organic matrices. This selective retention of the collagen scaffold can be significant to the adhesive performance of resin-based sealers and adhesive restorations because the uncovered collagen fibrils serve as substrates for hybrid layer growth and micromechanical interlocking [23]. The excellent maintenance of microhardness in the two materials studied, as shown by the superiority of MTAD and QMix, is one of the most clinically applicable results of this study. The concentration of citric acid in MTAD is rather low, along with doxycycline, an antimicrobial agent and a decreased form of the enzyme matrix metalloproteinase, which could provide some protective effect on the exposed collagen matrix against enzymatic breakdown [24]. In the past, it has been shown that the anti-collagenase effect of doxycycline can partially offset the demineralizing action of the citric acid component, resulting in net dentin structural integrity higher than that expected from the chelating capacity of the solution alone [25]. A lower concentration of EDTA in its reduced form, QMix, used together with chlorhexidine and a surfactant, is effective at chelation and presents minimal mineral depletion, owing to its optimized formulation concentration [26]. The gradual loss of microhardness from coronal to apical root levels across all groups reflects the long-known regional differences in dentin composition and tubular architecture. The apical dentin is said to be denser in tubules, less in tubular diameter, more sclerotic and with variation of mineral content than coronal root dentin [27]. The dentin matrix diffusion kinetics arising from these structural differences can generate varying demineralization patterns across different levels of the root and this may lead to varying patterns of apical ectodermal ridge and salivary gland diffusion. The distance-dependent character of irrigant penetration and chemical activity is also attested by the observation that the microhardness drop was always higher at 500 μm than at 1000 μm in the canal lumen, with the dentin next to the canal wall being the most vulnerable to the effect of irrigant [28]. When it comes to the issue of smear layer removal, it is possible to explain why QMix is better than all root canals: the formulation of this product includes a surfactant that decreases the tension among liquids and increases the wettability and the ability of the solution to penetrate the small spaces of the apical root canal and dentinal tubular system [29]. The surfactant agent helps create closer contact between the active chelating agents and the smear layer substrate, specifically in the apical third, where an anatomic limit restricts the delivery and exchange of conventional irrigants [30]. The similar molar removable effect of MTAD to that of the traditional NaOCl/EDTA protocol confirms the clinical use of this solution, as observed in other comparative assessments and the detergent component of this protocol (Tween 80) also improves the solution's spreading properties [31]. The low quality of the smear layer found in the CHX/EDTA group, specifically in the apical third, despite the presence of EDTA, may be related to the dynamics of interaction between chlorhexidine and the dentin substrate. It is known that Chlorhexidine forms a substantivity bond with dentin by a substantivity mechanism that is an electrostatic interaction between the cationic bisbiguanide molecule and the anionic dentin surface components [32]. This attachment can partially block tubule orifices and form a surface layer that hinders the latter effect of EDTA on the corresponding smear layer. Also, it has been reported that an insoluble precipitate forms when chlorhexidine is exposed to residual sodium hypochlorite or certain chelating agents, which may further undermine the effectiveness of debridement [33]. The relative decrease in the apical third of smear layer versus both coronal and middle thirds of the smear layer is a fact that can be attributed to a combination of factors such as lower irrigant volume exchange because of the small canal diameter, vapor lock effect which forms gas entrapment, increased smear layer density because of the increased contact with the instrumentation of the apical preparation and the nature of the difficulty in delivering irrigant solutions to the apical depth of the preparation [34]. Several limitations are associated with this study. Although the in vitro design allows standardized experimental conditions, it fails to fully simulate the clinical environment, including the presence of vital or necrotic pulp tissue, bacterial biofilms, periapical fluid dynamics, or the impact of body temperature on the solution's activity. The exposure times and volume were standardized and may not accurately reflect the variation in clinical irrigation practice. The long-term effects of microhardness changes in endodontic materials resulting from irrigant use were not evaluated and warrant further research [35].

## Conclusion:

All irrigation regimens significantly reduced radicular dentin microhardness, with NaOCl/EDTA causing the greatest loss, while QMix/MTAD balanced superior smear-layer removal with minimal dentin weakening. CHX/EDTA preserved hardness better than NaOCl protocols but showed inferior smear removal, particularly in the apical third, limiting its use as a standalone agent. QMix and MTAD emerge as optimal alternatives to conventional protocols, offering clinicians' superior cleaning efficacy while better preserving dentin structural integrity.

## Figures and Tables

**Table 1:** Vickers Microhardness Values (VHN) at 500 µm from Canal Lumen

**Group**	**Coronal Third**	**Middle Third**	**Apical Third**	**p-value (intra-group)**
Group 1 - NaOCl/EDTA	42.36 ± 3.84^a^	40.18 ± 4.12^a^	38.74 ± 4.56^a^	0.042*
Group 2 - NaOCl/Citric acid	43.92 ± 3.68^a^	41.46 ± 3.94^a^	39.28 ± 4.82^a^	0.028*
Group 3 - CHX/EDTA	48.54 ± 3.26^b^	46.82 ± 3.72^b^	43.16 ± 4.28^b^	0.003*
Group 4 - MTAD	51.28 ± 3.14^bc^	49.64 ± 3.48^bc^	46.52 ± 3.96^b^	0.006*
Group 5 - QMix	52.46 ± 2.92^c^	50.18 ± 3.26^c^	47.34 ± 4.14^b^	0.004*
Group 6 - Saline (Control)	62.84 ± 2.78^d^	61.52 ± 3.04^d^	58.96 ± 3.42^c^	0.012*
p-value (inter-group)	<0.001*	<0.001*	<0.001*	
*Statistically significant (p < 0.05);
Different superscript letters within
each column indicate statistically
significant differences (Tukey's HSD, p < 0.05)

**Table 2:** Vickers Microhardness Values (VHN) at 1000 µm from Canal Lumen

**Group**	**Coronal Third**	**Middle Third**	**Apical Third**	**p-value (intra-group)**
Group 1 - NaOCl/EDTA	52.14 ± 3.46^a^	50.28 ± 3.82^a^	47.62 ± 4.24^a^	0.018*
Group 2 - NaOCl/Citric acid	53.48 ± 3.28^a^	51.72 ± 3.64^a^	48.34 ± 4.48^a^	0.009*
Group 3 - CHX/EDTA	56.82 ± 3.14^b^	54.96 ± 3.42^b^	51.28 ± 3.86^b^	0.002*
Group 4 - MTAD	59.36 ± 2.94^bc^	57.48 ± 3.18^bc^	54.16 ± 3.62^bc^	0.001*
Group 5 - QMix	60.12 ± 2.72^c^	58.26 ± 3.08^c^	55.42 ± 3.74^c^	0.003*
Group 6 - Saline (Control)	63.18 ± 2.68^c^	61.84 ± 2.96^d^	59.28 ± 3.36^d^	0.008*
p-value (inter-group)	<0.001*	<0.001*	<0.001*	
*Statistically significant (p < 0.05);
Different superscript letters within each
column indicate statistically significant
differences (Tukey's HSD, p < 0.05)

**Table 3:** Smear layer removal scores - median (interquartile range)

**Group**	**Coronal Third**	**Middle Third**	**Apical Third**	**p-value (intra-group)**
Group 1 - NaOCl/EDTA	1.0 (1.0-2.0)^a^	2.0 (1.0-2.0)^a^	2.5 (2.0-3.0)^ab^	0.008*
Group 2 - NaOCl/Citric acid	1.5 (1.0-2.0)^a^	2.0 (1.0-2.0)^a^	3.0 (2.0-4.0)^b^	0.003*
Group 3 - CHX/EDTA	2.0 (1.0-2.0)^ab^	2.5 (2.0-3.0)^b^	3.5 (3.0-4.0)^bc^	0.001*
Group 4 - MTAD	1.0 (1.0-2.0)^a^	2.0 (1.0-2.0)^a^	2.0 (2.0-3.0)^a^	0.012*
Group 5 - QMix	1.0 (1.0-1.0)^a^	1.5 (1.0-2.0)^a^	2.0 (1.0-2.0)^a^	0.016*
Group 6 - Saline (Control)	5.0 (4.0-5.0)^c^	5.0 (5.0-5.0)^c^	5.0 (5.0-5.0)^d^	0.368
p-value (inter-group)	<0.001*	<0.001*	<0.001*	
*Statistically significant (p < 0.05);
Different superscript letters within
each column indicate statistically
significant differences (Mann-Whitney
U with Bonferroni correction, p < 0.05)
